# Ten simple rules for designing and conducting undergraduate replication projects

**DOI:** 10.1371/journal.pcbi.1010957

**Published:** 2023-03-16

**Authors:** David Moreau, Kristina Wiebels

**Affiliations:** School of Psychology and Centre for Brain Research, University of Auckland, Auckland, New Zealand; Carnegie Mellon University, UNITED STATES

## Abstract

Conducting a replication study is a valuable way for undergraduate students to learn about the scientific process and gain research experience. By promoting the evaluation of existing studies to confirm their reliability, replications play a unique, though often underappreciated, role in the scientific enterprise. Involving students early in this process can help make replication mainstream among the new generation of scientists. Beyond their benefit to science, replications also provide an invaluable learning ground for students, from encouraging the development of critical thinking to emphasizing the importance of details and honing research skills. In this piece, we outline 10 simple rules for designing and conducting undergraduate replication projects, from conceptualization to implementation and dissemination. We hope that these guidelines can help educators provide students with a meaningful and constructive pedagogical experience, without compromising the scientific value of the replication project, therefore ensuring robust, valuable contributions to our understanding of the world.

In scientific research, a replication is commonly defined as a study that is conducted using the same or similar methods as the original investigation, in order to evaluate whether consistent results can be obtained [1]. Often carried out by researchers independent from the original investigators, replications are designed to assess the robustness and generalizability of the original findings [2,3]. Beyond their clear scientific merit, replications are also a tremendously valuable way for undergraduate students to learn about the scientific process and gain research experience [4–6]. Conducting a replication study gives students a chance to acquire experience about how to design and manage a study, collect and analyze data, and report findings in a clear and concise manner. By promoting the evaluation of the methodology and results of the original study and considering whether the findings are supported by the data, replication projects also allow the development of critical thinking skills, together with an acute understanding of the importance of replicability in science [7,8].

However, replicating a study can also be a challenging and time-consuming task. One challenge comes from the fact that replication projects often require a high level of attention to detail and meticulous planning, as students must carefully follow the methodology of the original study in order to ensure that their replication is valid and accurate. This can be especially difficult if the original study used complex or specialized techniques, or if the materials or equipment needed to conduct the study are not readily available. In addition, replication projects may require a significant time commitment and resource investment, as students may need to collect and analyze large amounts of data in order to accurately replicate the original study.

When it comes to grappling with these challenges and making the most out of an undergraduate replication project, a few recommendations can go a long way. Below are 10 simple rules that can guide or facilitate replication research at the planning stage, while conducting research, and when preparing a completed replication study for publication. Although these rules are primarily intended for educators, we hope they can also provide important pointers for students embarking on a replication project. We provide an open-access questionnaire supervisors can use with their students to facilitate seamless implementation of these rules (available at https://github.com/davidmoreau/rep_checklist). Note that we focus our examples on the replication of empirical work; however, repeating computational or simulation work can also be a valuable way to engage undergraduate students in the scientific process, especially in fields where this kind of work is the norm. See [9–11] for more details on the distinction and practical examples.

## Rule 1: Clearly explain the purpose and importance of replication

Do not expect students to just know why replication is important. Many might, but even for those students the value of replication for science bears repeating [8,12]. Conveying the importance of replication can take many forms, but it often centers on 2 core aspects: the importance of replication within the scientific process, and its pedagogical value as part of students’ curriculum.

One way to emphasize how crucial replication is to the scientific process is by discussing how this type of work allows researchers to verify the results of previous studies and confirm that they are reliable and consistent [13–16]. Many students might not realize that published studies, despite having been peer-reviewed, still sometimes report results that are either simply erroneous, spurious, or based on poor methodological designs. In this context, correcting the scientific record is a fundamental endeavor, especially for scientific findings that are used to inform decisions or to guide the development of new theories or treatments. By replicating a study, researchers can ensure that the results are robust, thus helping build a cumulative, trustworthy body of knowledge.

Via the role it plays in addressing and correcting errors or biases in research, doing replication work also provides a great opportunity to highlight the human side of the scientific enterprise, and particularly how scientists may make mistakes or be influenced by their own biases, which can lead to flawed or biased results [17,18]. In this context, replication allows researchers to identify and correct these errors or biases, which helps improve the reliability and validity of scientific findings. More generally, replications present an unparalleled platform to develop critical thinking skills and improve students’ understanding of the scientific process by providing them with a hands-on approach to research.

## Rule 2: Choose the right study

When choosing a study to replicate, it is important to select a project that is appropriate for the student, yourself, and also given the resources available. For example, consideration of the specific skill level of your students is key. This means selecting a study that is not too complex or advanced for students to handle, given their current level of knowledge and experience. Choosing a study that is overly difficult or technical may result in frustration and difficulties for the students and could even lead to poor results or a lack of interest in the project. On the other hand, choosing a study that is too simple may not provide students with a meaningful learning opportunity or challenge them to think critically.

There are several factors to consider when determining whether a study is appropriate for the skill level of your students. These may include the complexity of the study’s methodology, the amount of data that needs to be collected and analyzed, the level of statistical or technical knowledge required to understand the study results, and the amount of time and resources that will be needed to complete the project. It may also be helpful to consider the interests and goals of your students, as well as their previous experience with research or similar projects. Have they conducted or participated in research before? Do they have experience writing up results? Do they already have basic or perhaps more advanced statistical skills? By carefully considering these factors, you can choose a study that is challenging yet achievable for your students and that will provide them with a valuable learning experience.

In addition to skill level, the availability of funding, equipment, and personnel, as well as the amount of time available to complete the study should also be factored in. This will help ensure that the study chosen is appropriate to the resources and time available and that it can be completed in a timely and efficient manner. To do this effectively, make sure you consider the scope of the original study and the methodology used, as these factors can have a significant impact on the resources and time required to complete the replication. For example, if the original study used complex statistical techniques or required specialized equipment, the replication may require additional resources and time that are not available given the constraints of the program or the institution.

Finally, not every study is worth replicating: A number of criteria have been proposed to maximize the benefit derived from a replication study, including via a consideration of the expected utility gain of the replication [19]. Here, it can be worth joining one of the large-scale consortia that advertise multi-lab replication work, as these often focus on replicating work that is known to be highly impactful in a field of study. Examples abound, but many calls get advertised on platforms such as the Center for Open Science—which has published calls for multi-lab replication projects in fields such as psychology, neuroscience, or cancer biology—or via more informal announcements on social media. The specifics of replication work is changing at a rapid pace [20], and so it is important to stay up-to-date with the literature to ensure discussions with students are current and constructive. By taking all these factors into account, you can propose a replication study that is appropriate and meaningful to students, and one that sets them on a path to success and fulfillment.

## Rule 3: Use preregistration to increase transparency and reduce bias

Planning ahead is essential to the success of a replication study. This often means preparing potential projects long before students join your lab or research group, for example, by consulting resources about good practices for designing replications [20] or applying for ethical approval via your institutional review board if appropriate. This way, students are able to start working on a replication study immediately after it has been selected, without further delay.

Once a clear plan has been made with the student, it is important to preregister it to increase transparency and reduce bias [21]. Preregistration refers to the practice of publicly sharing the research plan for a study, including the research question, hypotheses, design, measures, and analysis plan, before the study is conducted [22]. Preregistration can increase transparency by making the research plan for a study publicly available and allowing other researchers to evaluate and comment on it before the study is conducted (for example, with Registered Reports [23]; see also *Rule 10*). This can reduce bias by allowing researchers to identify and correct any potential problems or biases in the research plan before the study is conducted [24].

There are a number of platforms available to preregister: consider using the general-purpose Open Science Framework (OSF; https://osf.io/) or AsPredicted (https://aspredicted.org/) for studies that are not interventions or reviews. For most specific study types such as clinical trials or meta-analyses, specialized registries exist, such as ClinicalTrials.gov (https://clinicaltrials.gov/), the AEA RCT Registry (https://www.socialscienceregistry.org/), or the International Prospective Register of Systematic Reviews PROSPERO (https://www.crd.york.ac.uk/prospero/). By making their research plan publicly available on one of these platforms, researchers can improve transparency and lessen the influence of biases, helping improve the reliability and validity of the replication study results.

## Rule 4: Encourage open communication and collaboration with the original authors

It is often a good idea to encourage open communication and collaboration with the original authors. This means actively seeking out the original authors of the study and engaging in dialogue with them about your replication study. It is recommended to follow good practices in this process such as being respectful and professional, clearly explaining the purpose and goals of the replication, and asking for any relevant information or materials needed to accurately replicate the study. Students might not typically be well-versed in those aspects of academic life; fortunately, templates have been developed that can be adapted to fit specific purposes of the project [25]. It is also important to keep the authors informed throughout the replication process and to be open to their feedback and suggestions. Maintaining open communication with the original authors can help ensure the replication is conducted accurately and can also foster a positive and productive relationship between the replicating and original research teams.

Communication with the original authors can be beneficial for several reasons. First, it allows asking questions and clarifying any points that are unclear or confusing in the original study. Although the primary purpose of methods sections in journal articles is to allow understanding and reproducing procedures and analyses exactly, empirical evidence has indicated this is typically not the case [26]. As a result, direct communication with the original authors is often invaluable, as it can help better understand the study and replicate it more accurately. Conversations with the original authors can also facilitate the identification of any errors or mistakes in the original study that may have been overlooked or even lead to the sharing of new insights and perspectives, helping to advance scientific knowledge.

Finally, corresponding with the original authors about the replication can help build relationships and foster a sense of community within the scientific field. This can be especially beneficial for undergraduate students, as it can facilitate the development of new connections and help gain valuable mentorship and guidance from experienced researchers. When done well and in a considerate manner, this open process can help increase the visibility and reputation of your students within the scientific community, possibly leading to future collaborations and opportunities for them.

## Rule 5: Follow the original study’s methodology as closely as possible

For a replication project to be both valid and valuable, it is important to follow the original study’s methodology as closely as possible. Note that we assume here that the intent is to conduct a *direct* replication, as these are often better suited to undergraduate research projects given that they tend to be less complex and require fewer resources [20], but see [27] for a discussion of *conceptual* replication. Conducting a direct replication involves attempting to replicate the study as closely as possible, including using the same research design, measures, procedures, and data analysis techniques as the original study [20,28].

There are several reasons why it is essential to follow the original study’s methodology as closely as possible when conducting a replication study. One reason is that replication allows researchers to identify and correct errors or biases, and this function might be compromised if the replication protocol does not match the original. Another, perhaps more important reason stems from the primary purpose of replications, which is to verify the results of previous studies and confirm that they are reliable and consistent. In this context, if a replication departs substantially from the original, it is difficult to know what a discrepancy in findings would mean. Are the original study and the replication coming to a different conclusion because the original findings were not reliable? Or is it because of the change in methodology or poor implementation of the replication? Without abiding by the original protocol, the conclusions one can draw from a replication can be limited, especially when results are discrepant across studies. By replicating a study using the same methods as the original study, researchers can ensure that the results are robust and trustworthy.

Despite these potential challenges, some replication studies might deliberately opt to vary parameters, for example, by involving a different population (e.g., healthy versus clinical) or different measures (e.g., one measure of cognitive ability versus another) than in the original investigation. Those studies might be extremely valuable, but they need to be very carefully planned to yield insight, and often require a deeper understanding of the research question and of its subfield compared to direct replications. Because such thorough understanding takes time, undergraduate students often do not bring this kind of expertise to a project, so it typically falls upon the supervisor to design an adequate replication study. That said, such replication studies arguably have greater potential to spark new programs of research for students to pursue, which might be a factor to consider for those aiming for a career in science. More complex replication protocols might thus be more suited to more experienced graduate students, whose time commitments are usually greater.

## Rule 6: Clearly document all steps of the replication process

Although this advice is valid irrespective of the type of research project, clearly documenting all steps of the research process is particularly important for replications. This means keeping thorough and detailed records of all aspects of the study, including the research question, hypotheses, design, participants, measures, procedures, data collection and analysis, and results. By providing a detailed and transparent record of your study, students make sure other researchers will understand their methodology and will be able to evaluate the reliability and validity of the results.

Fortunately, many resources are available that can help document all steps of the project. First and foremost, the preregistration plan provides a guide for students to follow, making it clear when deviations from the intended protocol occur. Templates have been developed for researchers to use (see *Rule 3* for examples). Remember to convey to your students that deviations from intended protocols happen, even to experienced researchers, but that they should be clearly documented. Specifically, one should state what was different from the preregistration, the reason for deviating from the plan, and what was done, if anything, to address the deviation.

Second, it can help to set up a good communication workflow for your students, for example, with a project management platform (e.g., Trello) linked to a communication and collaboration tool (e.g., Slack) and a platform for live editing of text, code, or visualizations (e.g., Jupyter notebooks). Together, such workflow makes communication and collaboration seamless and can help prevent deviations before they occur. Within this workflow, encourage your students to keep a detailed lab or field notebook, using clear and consistent terminology, and including diagrams, tables, or other visual aids where necessary. Discussing good practices in documenting research might also provide an excellent opportunity to touch on reproducible protocols, including via computationally reproducible environments—aspects of research that are becoming more and more important and that we believe should be taught to young scientists [29].

Throughout the project, good documentation also enables you to review your student’s work. By keeping a clear and detailed record of the study, you can identify any areas where the student may have made mistakes or where they could have done things differently. This can be a valuable learning experience for undergraduate students, as it allows them to gain insight into the research process and to develop critical thinking skills. Furthermore, documentation also ensures that a replication is reproducible; this means that other researchers should be able to replicate the replication study using the information that was provided.

## Rule 7: Use appropriate statistical analyses

Using sound and adequate statistical analyses is key to extract the most out of replication data. Here, it is useful to distinguish between 2 different scenarios: one where statistical analyses have been decided for you, and one in which you have to come up with your own statistical plan with your student.

In the first scenario—common in multi-lab replication efforts—someone else (usually the lead team for the replication project) has developed a thorough statistical plan. In ideal cases, the statistical plan even comes with analysis code (e.g., R or Python). In such a scenario, analyses are typically straightforward, and can be as easy as making sure the data are in the right format for analysis, and that analytic outputs are interpreted correctly. Oftentimes, scripts for statistical analysis will be shared on platforms such as the Open Science Framework (osf.io) or GitHub (github.com), thus making it easy to access for all replicators, and seamless to push updates from the lead team if necessary. This kind of “ready-to-launch” project is well suited to students who have limited time available for the replication, for example, as part of a summer internship or for supervisors looking to get started but who have limited experience doing replication work.

In the second scenario we mentioned, the replication might either be large scale but not as readily structured, or it might be part of a more local effort, for example, at the scale of your own lab or research group. Under these circumstances, building an appropriate statistical plan is your responsibility and may involve a little more hands-on work. Oftentimes, using the analysis plan of the original study is the best course of action for the replication as well, yet these are not always available and may need to be inferred from the methods section of the original paper. In some cases, however, the original analysis plan might not be sound, either due to errors or because the statistical framework the original authors used is suboptimal.

Entire textbooks and a wealth of tutorials have been written about choosing the best statistical tests for a given research problem and data type; however, some specific resources might come in handy both for the supervisor and for the students involved in a replication. For example, it is worth considering issues such as statistical power in the context of replication [30–32], as well as best practices in the design and analysis of replication studies [33,34]. General resources about statistics in science can be beneficial to students who need a refresher on specific aspects of research methods; see for example [35] for an introductory online textbook on statistics, [36] for a great resource on the design of experiments and observational studies, [37] for a thorough guide covering various aspects of data analysis, or [38] for a primer on biostatistics using R. The following webpage (https://bookdown.org/home/tags/statistics/) includes these resources and many more to choose from depending on the needs of the project and of your students—all free and accessible to all.

## Rule 8: Collaboratively write a clear report of the findings

Writing a report of the findings that highlights the key points and implications of the study is another very important aspect of replication work. Crucially, this is key even (especially) if the original study did not replicate, or if the results turned out to not be significant. By presenting the results in a clear and easy-to-understand manner, you can help other researchers understand the methodology you and your student used and facilitate critical evaluation of the reliability and validity of the results.

For pedagogical reasons, it is often a good idea to collaborate with the student on the writing. This allows the student to gain hands-on experience in the writing process and to receive guidance and feedback from an experienced researcher. Scientific writing is a very specific type of exercise, and it can be greatly beneficial for students to see how the process unfolds for more experienced writers. Make sure you also point students toward resources to help them understand how to structure a scientific paper [39,40]. By working with you, students can also learn about the importance of clear and concise writing, as well as how to effectively communicate their findings. This process is also a great opportunity to discuss different perspectives and approaches to the research, which can broaden students’ understanding and critical thinking skills. Relatedly, writing up the research is also a good occasion to consider potential moderators or alternative explanations for the results, though if possible it is often useful to think about these at the onset of the project, so as to potentially incorporate additional, relevant data collection (e.g., moderating variables) as part of the replication plan.

Alongside the report, it might also be a good idea to post (publicly) the study materials and data, for example, on a repository like the Open Science Framework (osf.io). Make sure your students are aware of the specifics of data sharing [41,42], such as requesting permission from participants and from the local Ethics or Institutional Review Board approval. Even when permission is granted, it is important to ensure data is fully anonymized—this includes obvious identifiers such as first and last names, but also more subtle cues (e.g., extreme values or uncommon labels) that can give away participants’ identities [43–45]. Overall, this collaborative experience can be an extremely valuable opportunity for students to develop their research skills and gain a deeper understanding of the research process.

Finally, writing up a research report also facilitates clear communication about the ramifications of the study—by highlighting the key points and implications of the findings, students can learn to clearly communicate their work and its potential impact on the scientific community and beyond.

## Rule 9: Use appropriate citation practices

Essential to any academic write-up, using adequate citations is arguably even more important for a replication project. Obviously, this means properly citing the original study that was replicated, but also any other sources of information that were used in the replication [46]. Proper citation practices allow other researchers to find and evaluate the sources of information that were used in the replication [47] and give credit to the original authors and researchers. This helps recognize and acknowledge the contributions of the original authors and researchers, which is an important aspect of academic integrity. Relatedly, proper citations also contribute to avoiding plagiarism—a serious offense in the scientific community, though sometimes underappreciated by undergraduate students.

One way to foster proper citation practices is to provide clear guidelines on how to cite sources properly and direct students to relevant resources, such as citation style manuals (e.g., APA Publication Manual, MLA Style Manual, Chicago Manual of Style), online citation tools (e.g., Zotero, Mendeley, Endnote, Paperpile), or library databases (e.g., PubMed, ScienceDirect, Scopus, Web of Science). When appropriate, it can also be helpful to incorporate citation training into the curriculum more formally, for example, by making citation practices a central component of students coursework, with opportunities to practice and receive feedback. Finally, supervisors should foster an open and supportive environment where students feel comfortable asking questions and seeking clarification on citation practices—although the rules and conventions that govern citations are an inherent part of supervisors’ professional life, they often remain challenging for less seasoned students.

## Rule 10: Disseminate the findings

Sharing the findings of the replication study with the scientific community, either through publication or presentation, can contribute to the overall understanding of the original study and the scientific field. There are several ways to publish or present a replication study, including submitting it to a scientific journal, presenting it at a conference, or sharing it online (e.g., on a preprint repository such as arXiv, bioRxiv, medRxiv, or PsyArXiv). Note that some conferences actively seek out student presentations (see for an up-to-date list across areas of science and education: https://waset.org/student-conferences), while virtually all outlets that publish empirical work accept the submission of replication studies (some journals specifically encourage the submission of this type of study). Furthermore, many journals now accept Registered Reports [23,48,49]—a type of publication format where the methodology and planned analysis of a study are preregistered and peer-reviewed before data collection and analysis. The idea behind Registered Reports is to promote the transparency and reproducibility of scientific research by reducing the influence of publication bias and data-driven hypothesis testing [49]—goals that are well aligned with those of replication work generally. In a Registered Report, the peer-review process focuses on the study design, methodology, and data analysis plan, rather than the results, which are then reported regardless of their significance or outcome. Registered Reports are increasingly becoming a preferred publication format in various fields of research and provide an opportunity for researchers to promote rigorous and transparent scientific practices [48]. The Center for Open Science maintains a list of journals who accept the submission of Registered Reports (https://www.cos.io/initiatives/registered-reports).

Irrespective of the format, publishing or presenting the findings of the replication study is important for several reasons. Beyond enabling other researchers to review and evaluate the replication study, disseminating the results helps students contribute to the scientific community. This can be especially meaningful for undergraduate students, as it allows them to make a meaningful contribution to become a part of the larger scientific community. By improving the visibility and impact of a study, disseminating results can be especially important for undergraduate students, as it helps raise their profile and increase their career prospects within the scientific community [50].

In addition, publishing a paper with an experienced supervisor can help students gain a deeper understanding of the peer-review process and its significance. They can learn about the criteria used by reviewers to evaluate papers, the types of feedback and comments received, and the process of making revisions based on the feedback. This experience can help students appreciate the role of peer-review in improving the quality and credibility of scientific research and develop a better understanding of how to write and present their findings effectively to meet the standards of the scientific community.

Finally, working toward publication with an experienced supervisor gives students a chance to learn about the ethical and legal aspects of scientific publishing. This includes understanding the principles of plagiarism, data fabrication, and misconduct, and how to avoid these unethical practices. Students can learn about the importance of obtaining informed consent from study participants, properly citing sources, and obtaining appropriate permissions for using images and other copyrighted materials. They can also develop awareness for how to properly report conflicts of interest and ensure the transparency and accuracy of their research findings. Understanding the ethical and legal aspects of scientific publishing is critical to maintaining the integrity and reputation of the scientific community, and students can benefit greatly from learning these principles while working with their supervisor on a publication.

## Conclusion

Conducting a replication study with undergraduates can be a valuable and rewarding way for students to learn about the scientific process and gain research experience. Replication studies provide an accessible entry point to the world of scientific research, while at the same time, engaging them in the laudable process of verifying previous results to confirm their robustness and reliability. Overall, this process helps build confidence in the scientific community and advance our understanding of the world around us. By following the guidelines outlined in this article, we hope educators and students can increase the likelihood that their replication work will not only constitute an enjoyable learning experience, but will also make a meaningful, high-quality contribution to the scientific record.

## References

[1] Jeffreys H. Scientific Inference, Third edition. 3rd ed. Cambridge University Press; 1974.

[2] Camerer CF, Dreber A, Holzmeister F, Ho T-H, Huber J, Johannesson M, et al. Evaluating the replicability of social science experiments in Nature and Science between 2010 and 2015. Nat Hum Behav. 2018;2:637–644. doi: 10.1038/s41562-018-0399-z 31346273

[3] Errington TM, Mathur M, Soderberg CK, Denis A, Perfito N, Iorns E, et al. Investigating the replicability of preclinical cancer biology. Elife. 2021:10. doi: 10.7554/eLife.71601 34874005 PMC8651293

[4] Plucker JA, Makel MC. Replication is important for educational psychology: Recent developments and key issues. Educ Psychol. 2021;56:90–100.

[5] Cai J, Morris A, Hohensee C, Hwang S, Robison V, Hiebert J. The Role of Replication Studies in Educational Research. J Res Math Educ. 2018;49:2–8.

[6] Frank MC, Saxe R. Teaching Replication. Perspect Psychol Sci. 2012;7:600–604. doi: 10.1177/1745691612460686 26168118

[7] Simons DJ. The Value of Direct Replication. Perspect Psychol Sci. 2014;9:76–80. doi: 10.1177/1745691613514755 26173243

[8] Nosek BA, Errington TM. What is replication? PLoS Biol. 2020;18:e3000691. doi: 10.1371/journal.pbio.3000691 32218571 PMC7100931

[9] Lohmann A, Astivia OLO, Morris TP, Groenwold RHH. It’s time! Ten reasons to start replicating simulation studies. Front Epidemiol. 2022:2. doi: 10.3389/fepid.2022.973470 PMC10911016 38455335

[10] Hauke J, Achter S, Meyer M. Theory development via replicated simulations and the added value of standards. J Artif Soc Soc Simul. 2020:23. doi: 10.18564/jasss.4219

[11] Plesser HE. Reproducibility vs. Replicability: A Brief History of a Confused Terminology. Front Neuroinform. 2017;11:76. doi: 10.3389/fninf.2017.00076 29403370 PMC5778115

[12] Nakagawa S, Parker TH. Replicating research in ecology and evolution: feasibility, incentives, and the cost-benefit conundrum. BMC Biol. 2015;13:88. doi: 10.1186/s12915-015-0196-3 26510635 PMC4624660

[13] Klein RA, Vianello M, Hasselman F, Adams BG, Adams RB, Alper S, et al. Many Labs 2: Investigating Variation in Replicability Across Samples and Settings. Adv Methods Pract Psychol Sci. 2018;1:443–490.

[14] Ebersole CR, Atherton OE, Belanger AL, Skulborstad HM, Allen JM, Banks JB, et al. Many Labs 3: Evaluating participant pool quality across the academic semester via replication. J Exp Soc Psychol. 2016;67:68–82.

[15] The ManyBabies Consortium. Quantifying Sources of Variability in Infancy Research Using the Infant-Directed-Speech Preference. Adv Methods Pract Psychol Sci. 2020;3:24–52.

[16] Nosek BA, Alter G, Banks GC, Borsboom D, Bowman SD, Breckler SJ, et al. Promoting an open research culture. Science. 2015;348:1422–1425. 26113702 10.1126/science.aab2374 PMC4550299

[17] Simonsohn U, Nelson LD, Simmons JP. P-curve: a key to the file-drawer. J Exp Psychol Gen. 2014;143:534–547. doi: 10.1037/a0033242 23855496

[18] Kerr NL. HARKing: hypothesizing after the results are known. Pers Soc Psychol Rev. 1998;2:196–217. doi: 10.1207/s15327957pspr0203_4 15647155

[19] Isager PM, van Aert RCM, Bahník Š, Brandt MJ, DeSoto KA, Giner-Sorolla R, et al. Deciding what to replicate: A decision model for replication study selection under resource and knowledge constraints. Psychol Methods. 2021. doi: 10.1037/met0000438 34928679

[20] Brandt MJ, IJzerman H, Dijksterhuis A, Farach FJ, Geller J, Giner-Sorolla R, et al. The Replication Recipe: What makes for a convincing replication? J Exp Soc Psychol. 2014;50:217–224.

[21] Nosek BA, Ebersole CR, DeHaven AC, Mellor DT. The preregistration revolution. Proc Natl Acad Sci U S A. 2018;115:2600–2606. doi: 10.1073/pnas.1708274114 29531091 PMC5856500

[22] Bakker M, Veldkamp CLS, van Assen MALM, Crompvoets EAV, Ong HH, Nosek BA, et al. Ensuring the quality and specificity of preregistrations. PLoS Biol. 2020;18:e3000937. doi: 10.1371/journal.pbio.3000937 33296358 PMC7725296

[23] Henderson EL, Chambers CD. Ten simple rules for writing a Registered Report. PLoS Comput Biol. 2022;18:e1010571. doi: 10.1371/journal.pcbi.1010571 36301802 PMC9612468

[24] Dirnagl U. Preregistration of exploratory research: Learning from the golden age of discovery. PLoS Biol. 2020;18:e3000690. doi: 10.1371/journal.pbio.3000690 32214315 PMC7098547

[25] Moreau D, Gamble B. Conducting a meta-analysis in the age of open science: Tools, tips, and practical recommendations. Psychol Methods. 2022;27:426–432. doi: 10.1037/met0000351 32914999

[26] Errington TM, Denis A, Perfito N, Iorns E, Nosek BA. Challenges for assessing replicability in preclinical cancer biology. Elife. 2021:10. doi: 10.7554/eLife.67995 34874008 PMC8651289

[27] Schmidt S. Shall we Really do it Again? The Powerful Concept of Replication is Neglected in the Social Sciences. Rev Gen Psychol. 2009;13:90–100.

[28] Nosek BA, Errington TM. Making sense of replications. Elife. 2017:6. doi: 10.7554/eLife.23383 28100398 PMC5245957

[29] Wiebels K, Moreau D. Leveraging Containers for Reproducible Psychological Research. Adv Methods Pract Psychol Sci. 2021;4:25152459211017853.

[30] Jiang W, Yu W. Power estimation and sample size determination for replication studies of genome-wide association studies. BMC Genomics. 2016;17(Suppl 1):3. doi: 10.1186/s12864-015-2296-4 26818952 PMC4895704

[31] van Zwet EW, Goodman SN. How large should the next study be? Predictive power and sample size requirements for replication studies. Stat Med. 2022;41:3090–3101. doi: 10.1002/sim.9406 35396714 PMC9325423

[32] Piper SK, Grittner U, Rex A, Riedel N, Fischer F, Nadon R, et al. Exact replication: Foundation of science or game of chance? PLoS Biol. 2019;17:e3000188. doi: 10.1371/journal.pbio.3000188 30964856 PMC6456162

[33] Bonett DG. Design and Analysis of Replication Studies. Organ Res Methods. 2021;24:513–529.

[34] Skol AD, Scott LJ, Abecasis GR, Boehnke M. Joint analysis is more efficient than replication-based analysis for two-stage genome-wide association studies. Nat Genet. 2006;38:209–213. doi: 10.1038/ng1706 16415888

[35] Cappiello L. Introduction to Statistics. [cited 2023 Jan 31]. Available from: https://bookdown.org/lgpcappiello/introstats/.

[36] Taback N. Design and Analysis of Experiments and Observational Studies using R. [cited 2023 Jan 31]. Available from: https://designexptr.org/.

[37] Nguyen M. A Guide on Data Analysis. Bookdown; [cited 2023 Jan 31]. Available from: https://bookdown.org/mike/data_analysis/.

[38] A primer for biostatistics in R. [cited 2023 Jan 31]. Available from: https://bookdown.org/cj4nature/rstats4bio/.

[39] Mensh B, Kording K. Ten simple rules for structuring papers. PLoS Comput Biol. 2017;13:e1005619. doi: 10.1371/journal.pcbi.1005619 28957311 PMC5619685

[40] Eckhoff J. How to write an abstract. In: Springer Nature [Internet]. 18 Jan 2019 [cited 2023 Jan 31]. Available from: https://chemistrycommunity.nature.com/posts/43071-how-to-write-an-abstract.

[41] Wagenmakers E-J, Sarafoglou A, Aarts S, Albers C, Algermissen J, Bahník Š, et al. Seven steps toward more transparency in statistical practice. Nat Hum Behav. 2021;5:1473–1480. doi: 10.1038/s41562-021-01211-8 34764461

[42] Wilkinson MD, Dumontier M, Aalbersberg IJJ, Appleton G, Axton M, Baak A, et al. The FAIR Guiding Principles for scientific data management and stewardship. Sci Data. 2016;3:160018. doi: 10.1038/sdata.2016.18 26978244 PMC4792175

[43] El Emam K, Rodgers S, Malin B. Anonymising and sharing individual patient data. BMJ. 2015;350:h1139. doi: 10.1136/bmj.h1139 25794882 PMC4707567

[44] Keerie C, Tuck C, Milne G, Eldridge S, Wright N, Lewis SC. Data sharing in clinical trials—practical guidance on anonymising trial datasets. Trials. 2018;19:25. doi: 10.1186/s13063-017-2382-9 29321053 PMC5763739

[45] Haselgrove C, Poline J-B, Kennedy DN. A simple tool for neuroimaging data sharing. Front Neuroinform. 2014;8:52. doi: 10.3389/fninf.2014.00052 24904398 PMC4033259

[46] Penders B. Ten simple rules for responsible referencing. PLoS Comput Biol. 2018;14:e1006036. doi: 10.1371/journal.pcbi.1006036 29649210 PMC5896885

[47] Masic I. The importance of proper citation of references in biomedical articles. Acta Inform Med. 2013;21:148–155. doi: 10.5455/aim.2013.21.148-155 24167381 PMC3804522

[48] Chambers CD, Tzavella L. The past, present and future of Registered Reports. Nat Hum Behav. 2022;6:29–42. doi: 10.1038/s41562-021-01193-7 34782730

[49] Chambers CD. Registered reports: a new publishing initiative at Cortex. Cortex. 2013;49:609–610. doi: 10.1016/j.cortex.2012.12.016 23347556

[50] Allen C, Mehler DMA. Open science challenges, benefits and tips in early career and beyond. PLoS Biol. 2019;17:e3000246. doi: 10.1371/journal.pbio.3000246 31042704 PMC6513108

